# Revisiting Skull Metastases of Prostate Cancer at Prostate-Specific Membrane Antigen (PSMA) Positron Emission Tomography/Computed Tomography Era: PSMA Uptake Characteristics and Oncological Outcomes

**DOI:** 10.5152/tud.2025.24164

**Published:** 2025-03-06

**Authors:** Baris Esen, Okan Falay, Kayhan Tarim, Hulya Seymen, Mert Kilic, Sevil Bavbek, Yakup Kordan, Mehmet Onur Demirkol, Derya Tilki, Tarik Esen

**Affiliations:** 1 Department of Urology, Koç University Faculty of Medicine, Istanbul, Türkiye; 2 Department of Nuclear Medicine, Koç University Faculty of Medicine, Istanbul, Türkiye; 3 Department of Urology, VKF American Hospital, Istanbul, Türkiye; 4 Department of Medical Oncology, VKF American Hospital, Istanbul, Türkiye; 5 Department of Urology, University Medical Center Hamburg-Eppendorf Faculty of Medicine, Hamburg, Germany; 6 Martini-Klinik Prostate Cancer Center, University Hospital Hamburg-Eppendorf, Hamburg, Germany

**Keywords:** PET, prostate cancer, PSMA, skull metastasis

## Abstract

**Objective:**

We aimed to evaluate prostate-specific membrane antigen (PSMA) uptake characteristics and the oncological outcomes in patients with skull metastases.

**Methods:**

The records of 345 serial PSMA positron emission tomography (PET)/computed tomography (CT) scans of 96 patients with metastatic prostate cancer (PCa) were evaluated retrospectively. Skull bone metastasis was detected in 18 patients (18/96, 18.7%), with a mean age of 72.4 ± 9.1 years, and in 40 PSMA PET/CT scans (40/345, 11.6%). Involved skull bones, PSMA uptake characteristics, and CT counterparts of metastatic lesions were centrally reviewed. Prostate specific antigen (PSA) levels at the time of skull metastasis detection and PSMA-detected other metastatic lesions were recorded.

**Results:**

All patients with a skull metastasis showed multiple other metastatic bone lesions, and 6 (33.3%) had visceral metastasis. Seven (38.9%) patients had solitary skull lesions, whereas 11 (61.1%) had multiple skull metastases. Twenty-two out of 37 (59.5%) metastatic lesions had no CT counterpart. The median SUVmax was significantly higher in metastatic lesions with a CT counterpart (median 9.09 vs. 4.63, *P *= .018). At a median follow-up of 23.4 mo (interquartile range [IQR] 8.7-34.1) after detection of skull metastasis, 5 out of 11 (45.5%) hormone-sensitive and all castration-resistant patients died of PCa. The median survival of patients with castration-resistant disease was 9.92 months.

**Conclusion:**

The majority of PSMA-detected skull metastases did not show a CT counterpart, which may explain why skull metastases were rarely detected before the PSMA PET-era. In high-volume metastatic prostatic cancer cases, ^68^Ga-PSMA PET/CT imaging field including the vertex, may enhance the accuracy in detecting tumor extent and metabolic tumor volume.

**Main Points**

- Prostate-specific membrane antigen PET/CT may be detecting skull metastasis of PCa more frequently than conventional imaging.
- Skull metastasis of PCa is associated with high metastatic burden, and skip metastasis to skull bones seems to be infrequent.
- Prostate-specific membrane antigen PET/CT scans should be performed from the vertex to the toe for accurate evaluation of tumor extent and tumor volume.

## Introduction

Prostate cancer (PCa) is the second most commonly diagnosed cancer in men, with an estimated 1.4 million new cases worldwide in 2020.^1^ Although only 3% of patients with PCa have metastatic disease at initial diagnosis, 26.6% of patients who underwent radical prostatectomy for localized disease had metastatic progression along the course of the disease in the SPCG-4 trial.^2,3^ Metastatic progression represents the most common cause of cancer-associated morbidity and mortality.^4^ The most common sites of PCa metastasis are bones (84%), distant lymph nodes (LNs) (10.6%), liver (10.2%), and lungs (9.1%).^5^ Although PCa metastasis to axial bones is frequently observed, metastases to skull bones are considered rare, comprising less than 2% of all metastases of PCa.^6^ To date, even the largest series of patients with skull metastases of PCa included 8 to 11 patients.^7,8^ The prognosis of patients with skull metastasis was traditionally known to be poor, with a median survival of 1 year. A more recent meta-analysis of patients with skull-base metastases revealed a median survival of 21 months for patients with PCa.^9^

Positron emission tomography (PET) using prostate-specific membrane antigen (PSMA)-based tracers has been shown to be superior in detecting metastatic sites compared with conventional imaging at primary staging and imaging in biochemical recurrence (BCR) after radical prostatectomy.^10,11^ Prostate-specific membrane antigen PET/computed tomography (CT) has demonstrated higher sensitivity and specificity than ^99m^Tc-MDP bone scan in detecting bone metastasis.^12^ The superior diagnostic accuracy of PSMA PET/CT may result in an increased rate of detection of skull metastases of PCa. Therefore, current knowledge about the incidence and prognosis of PCa skull metastases should be challenged in the PSMA PET era. Herein, we aimed to investigate the PSMA uptake characteristics and the oncological outcomes in patients with PCa skull metastases detected by PSMA PET/CT.

## Material and Methods

In our previously published article, 345 serial PSMA PET scans performed on 96 patients with PSMA PET-detected metastatic PCa between January 2014 and June 2022 were evaluated to investigate the diagnostic performance of PSMA PET when monitoring metastatic prostate cancer (mPCa).^13^ Following approval from the Ethics Committee of Koç University (Approval no.: 2024.312.IRB2.141, Date: 24.09.2024 and obtaining written informed consent from all patients, these PSMA PET scans were retrospectively analyzed for the presence of skull bone metastases. Overall, skull bone metastasis was detected in 18 patients (18/96, 18.7%) and 40 PSMA PET/CT scans (40/345, 11.6%). In patients with multiple PSMA PET scans showing a skull metastasis, the initial PSMA PET scan with a skull metastasis was evaluated to describe the PSMA PET characteristics of the skull metastasis(es). The reason for performing the baseline scan with a skull metastasis was recorded in all cases (primary staging, imaging of BCR, or treatment monitoring). The BCR was defined as a serum PSA of ≥0.2 ng/mL above PSA nadir after radical prostatectomy and a PSA increase of ≥2 ng/mL above PSA nadir after radiotherapy. The PSMA PET scans performed for treatment monitoring were conducted in patients who underwent a baseline PSMA PET scan and a PSMA PET scan after receiving systemic treatment, any form of metastasis-directed therapy, and/or radiotherapy to the prostate for mPCa. All patient and tumor characteristics, such as age, PCa Gleason score, previously received treatment at the time of localized disease (radical prostatectomy/radiotherapy/none), and the metastatic stage (systemic treatments, metastasis-directed therapies, or treatment to primary) were recorded. The serum PSA level at the time of detection of skull metastasis was recorded. In all cases, metastatic sites other than skull bones (prostate, bone metastasis other than skull bones, visceral metastasis) were noted.

### Imaging Protocol and Evaluation of Prostate-Specific Membrane Antigen Positron Emission Tomography Parameters

A ^68^Ga-PSMA-11 dose of 2 mega becquerels (MBq) per kilogram up to 185 MBq was administered intravenously following a fasting period of at least 4 hours. Patients were advised to empty their bladder just before initiating the PET scan. Early images were captured at 45 minutes, and late abdominal images at 90 minutes post-injection. Additionally, a low-dose CT scan was conducted for attenuation correction. Subsequent cross-calibration was carried out to enhance the comparability of quantitative parameters. Computed tomography counterparts were recorded for all skull metastases. The spatial resolution of PET scanners (full width at half-maximum) was 4-6 mm. All externally performed PSMA PET scans were centrally reviewed by nuclear medicine specialists experienced in PSMA PET reporting, and technically unacceptable scans were excluded.

### Statistical Analysis

Statistical analyses were performed using SPSS version 24.0 (IBM SPSS Corp.; Armonk, NY, USA). Descriptive statistics for continuous parameters included mean ± standard deviation or median and interquartile range (IQR), while percentages were utilized for categorical parameters. The Mann–Whitney *U* test was utilized to compare the median values between groups. Kaplan–Meier survival estimates were utilized to evaluate overall survival rates. All tests were two-sided, and a *P*-value of less than .05 was considered statistically significant.

## Results

Skull metastasis was detected with PSMA PET/CT during primary staging in 6 (33%) and secondary staging in 12 (67%) patients. The median age and the median serum PSA at the time of skull metastasis were 72.4 years (IQR: 66.6-77.2) and 14.4 ng/dL (IQR: 5.03-133), respectively. The skull metastases were detected during primary staging in 6 patients, at the BCR imaging in 3 cases, and during the treatment monitoring of metastatic disease in 9 patients. Demographic and clinical characteristics of patients with skull metastases are provided in Table 1. Eleven out of 18 patients had metastatic hormone-sensitive PCa when skull metastasis was detected, whereas 7 patients had castration-resistant disease. All patients with skull metastasis showed multiple other metastatic bone lesions, and 6 (33.3%) had visceral metastasis. Supradiaphragmatic LN metastasis was present in 9 patients (50%). A total of 37 metastatic lesions at skull bones were detected. Seven (38.9%) patients had solitary skull lesions, while 11 (61.1%) had multiple skull metastases (Table 2).

**Figure 1.**
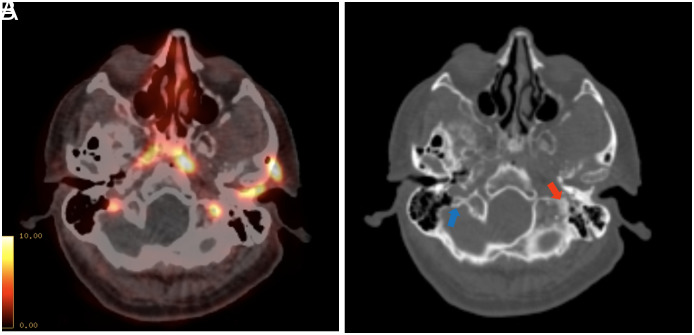
A, B. A 66 year-old patient underwent PSMA PET/CT due to a rise in serum PSA to 3.1 ng/dL post-radical prostatectomy. The PSMA PET scan revealed widespread metastatic disease at LN and multiple bones including left occipital and bilateral sphenoid metastases. (A) Axial fusion image depicting 2 PSMA-expressing metastatic tumors in the right and left sphenoid bones. (B) Standard CT image revealing the metastatic lesion on the right side (SUVmax: 5.63) does not have a CT counterpart while the metastatic lesion at the left sphenoid bone (SUVmax: 14.03) exhibits an osteosclerotic CT counterpart.

The median SUVmax of skull metastases was 5.35 (IQR: 3.37-9.03) while the SUVmax of other metastatic lesions varied between 2.2 and 72. None of the cases exhibited the skull as the site for metastatic lesions with the highest PSMA uptake (SUVmax). Twenty-two out of 37 (59.5%) metastatic lesions did not have any CT counterpart (Figure 1A and B). The median SUVmax was significantly higher in metastatic lesions with a CT counterpart (median 9.09 vs. 4.63, *P *= .018). At a median follow-up of 23.4 months (IQR 8.7-34.1) after detection of skull metastasis, 5 out of 11 (45.5%) hormone-sensitive and all castration-resistant patients died of PCa. The median survival of patients with castration-resistant disease was 9.92 months.

## Discussion

The development of bone metastases occurs as a result of a complex multi-step process between tumor cells and bone microenvironment.^14^ Despite bone being a common site of systemic metastases, the true incidence of skull metastases is underestimated since the clinical presentation of skull metastases frequently remains subtle until dura mater is invaded or local symptoms occur due to the compression of dural sinuses or cranial nerves, or form a mass large enough to cause cosmetic problems.^15^ A meta-analysis including 279 cases with skull metastasis revealed that the most common malignancies metastasizing to skull bones are PCa (38.5%) and breast cancer (20.9%). Skull metastases are traditionally diagnosed using conventional imaging modalities such as magnetic resonance imaging (MRI), CT, and bone scans. As one of the most distant axial bones, skull metastases were previously known as a late presentation of PCa and to be associated with poor prognosis with a median survival of 1 year. A meta-analysis reported a median survival of 21 months in patients with skull metastases of PCa.^9^ A recent study investigating 107 patients with bone metastasis (of whom 26 had skull metastasis) of PCa found that skull metastasis is associated with more aggressive disease and poor prognosis.^16^ It is important to note that the above-mentioned data about the incidence and oncological outcomes relies on patients with skull metastases diagnosed by conventional imaging or FDG PET/CT and needs to be challenged in the era of PSMA PET/CT which is a novel and superior diagnostic modality to detect distant metastasis in patients with PCa.

A prospective multicenter study has shown a higher diagnostic accuracy for PSMA PET/CT to detect LNs and distant metastatic lesions compared to conventional imaging techniques (CT and bone scan) in primary staging of PCa.^10^ Furthermore, PSMA PET/CT is the most sensitive diagnostic modality in the imaging of BCR, especially in patients with low PSA values (<0.5 ng/dL).^17^ Therefore, PSMA PET has a very prominent role in the primary staging and imaging of BCR in the current guidelines.^18^ Although PSMA PET is not routinely used in treatment monitoring currently, it is anticipated to have a significant role in the near future in evaluating treatment response, given the superior diagnostic accuracy to detect metastatic lesions as more data accumulates about the impact of PSMA PET on oncological outcomes.^19^ Our previous study revealed that PSMA PET can show metastatic lesions even in very low PSA values, and PSMA response might better representing tumor heterogeneity and the discordant response of different metastatic sites to systemic treatment compared with PSA response in the follow-up metastatic PCa.^13^ In our results, skull bone metastasis was detected in 18 patients (18/96, 18.7%) and 40 PSMA PET/CT scans (40/345, 11.6%), suggesting that skull metastases are observed much more frequently than previously reported incidence rates in the literature when serial PSMA PET/CT scans were performed to monitor treatment response in patients with mPCa. It is also observed that skull metastases were not necessarily detected in the late stages of PCa. Skull metastasis was first detected in primary staging or the imaging of BCR in half of the cases (9 out of 18 cases). Moreover, 11 out of 18 patients had hormone-sensitive disease when skull metastasis was detected. Similar to previous reports, the median survival of patients with skull metastases at the castration-resistant state was 9.92 months. However, the median survival was not reached at a median follow-up of 23.4 months after the detection of skull metastasis at the hormone-sensitive stage. The intriguing question of whether skull metastases are associated with poor oncological outcomes different from other bone metastases or the volume of metastatic disease remains to be investigated in the era of PSMA PET/CT. As one might notice from Table 1, it was very interesting to observe the development of metastatic disease, including skull bones, in a patient where the pathological examination of the radical prostatectomy specimen revealed GG1 disease (pT3bN0, Gleason score 3 + 3). However, it contradicts previous large series that extraprostatic extension in GG1 disease is extremely rare, while seminal vesicle invasion, lymph node metastasis, or distant metastasis never occurs.^20,21^ We can only think of the possibility of an error in the pathological evaluation of the radical prostatectomy specimen (performed in another center in 2003) to explain this issue.

Isolated skull metastasis or occult disease presenting with skull metastasis was previously reported in the literature.^22,23^ However, in our experience, skull metastases were associated with high-volume metastatic disease; all patients had multiple other bone metastases, 9 patients had supradiaphragmatic LN, and 6 patients had visceral metastasis(es), and no isolated skull metastases were observed. Again, the majority of patients had multiple lesions at the skull at diagnosis. Interestingly, 22 out of 37 lesions did not have any osteolytic or osteoblastic CT counterpart, and those with a CT counterpart had significantly higher SUVmax than those without a CT counterpart. Although low-dose CT scans performed during PSMA PET scans are not optimal for evaluating the CT counterpart of the metastatic lesions, they may provide an explanation for low detection rates of skull metastases by conventional imaging techniques.

To date, several PSMA PET response criteria have been proposed to define progression or response to treatment in patients receiving systemic treatment for mPCa. These criteria define progression as the development of new metastatic lesions or an increase in PSMA PET tumor volume.^24,25^ Similar to our findings, a recent study investigated metastatic bone lesions in 388 patients who underwent a vertex-to-toe total body PSMA PET/CT for PCa. Eighteen patients had metastatic bone lesions above the superior orbital ridge.^26^ Considering the high skull metastasis detection rates in the PSMA PET era**, **a vertex-to-toe total body PSMA PET/CT should be performed to better depict the tumor extent and to evaluate treatment response using standardized PSMA PET response criteria.

This study has several strengths and limitations. The main strength of this study is the standardized evaluation of PSMA PET scans and CT counterparts by an experienced nuclear medicine specialist. As for limitations, first, this is a retrospective study with its inherent limitations. Secondly, CT counterparts were evaluated using low-dose CT scans performed during PSMA PET/CT for attenuation purposes, which is not an optimally performed CT to evaluate bone metastasis. An additional bone scan or MRI was not performed to verify whether these lesions would have been detected by conventional imaging.

PSMA PET-detected skull metastasis may be more frequently observed than with conventional imaging. Skull metastasis was found to be associated with high metastatic burden. ^68^Ga-PSMA PET/CT imaging field including the vertex, may increase the accuracy of detecting tumor extent and tumor volume in cases with high-volume metastatic prostatic cancer.

## Figures and Tables

**Table 1.** Demographic and Clinical Characteristics of Patients with Skull Metastases

Parameter	
Number of patients, n	18
Age (years), median (IQR)	72.4 (66.6-77.2)
Prior local treatment	
Radical prostatectomy	6 (33.3%)
Radiotherapy to prostate	2 (11.1%)
Gleason score at PCa diagnosis*	
ISUP grade 1	1 (5.6%)
ISUP grade 2	1 (5.6%)
ISUP grade 3	5 (27.8%)
ISUP grade 4	2 (11.1%)
ISUP grade 5	8 (44.4%)
Prior pelvic lymph node dissection, n (%)	5 (25%)
pN0	4/5 (80%)
pN1	1/5 (20%)
The reason for initial PSMA PET scan, n (%)	
Primary staging	6 (33%)
Biochemical recurrence imaging	3 (17%)
Follow-up of metastatic disease	9 (50%)
PSMA-avid lesion sites	
Lymph node metastasis	15 (83%)
Bone metastasis	18 (100%)
Visceral metastasis	6 (33%)

*Gleason score data is not available in one patient.

ISUP: The International Society of Urological Pathology.

**Table 2.** Characteristics of Detected Skull Metastases, Received Treatments, and Oncological Outcomes

Patient	Age*	Reason for PSMA	Involved Skull Bone(s)	Other Metastatic Sites	Castration Status	PSA (ng/dL)	Treatment	Follow-Up (mo)	Latest Situation
1	75	Primary staging	R temporal, occipital	Bones, LN, and visceral (pulmonary)	Hormone sensitive	1066	ADT	34.07	Alive with disease
2^ψ^	75	Primary staging	Bilateral parietal, L frontoparietal, R sphenoid, L mastoid process	Bones, LN, and visceral (pulmonary)	Hormone sensitive	5.16	Cisplatin+Etoposide	7.23	Cancer-specific mortality
3	72	Primary staging	L mandible, occipital, sphenoid	Bones and LN	Hormone sensitive	5000	ADT+Palliative RT / ADT+Enzalutamide	22.74	Alive with disease
4	67	Primary staging	L parietal, R mandible	Bones and LN	Hormone sensitive	14.4	ADT+MDT / ADT+Abiraterone	32.66	Cancer-specific mortality
5	69	Primary staging	Occipital	Bones and LN	Hormone sensitive	22.5	ADT+Docetaxel / RLT	59.96	Cancer-specific mortality
6	90	Primary staging	L parietal, R frontal, occipital	Bones, LN, and visceral (pulmonary and corpu cavernosum)	Hormone sensitive	1318	ADT	42.22	Alive with disease
7	66	BCR imaging	Occipital and sphenoid	Bones and LN	Hormone sensitive	3.1	ADT+Docetaxel / ADT+Abiraterone	13.63	Alive with disease
8	67	BCR imaging	Occipital	Bones, LN, and visceral (pulmonary)	Hormone sensitive	1.9	Docetaxel / Abiraterone / MDT / RLT	16.23	Cancer-specific mortality
9	78	BCR imaging	R temporal	Bones	Hormone sensitive	7.17	ADT	29.08	Alive with complete response
10	70	Metastatic disease follow-up	Clivus	Bones and LN	Hormone sensitive	0.3	ADT+MDT	8.54	Cancer-specific mortality
11	65	Metastatic disease follow-up	Right temporal	Bones and LN	Hormone sensitive	NA	ADT+ Docetaxel / MDT / ADT	38.34	Alive with disease
12	77	Metastatic disease follow-up	Right sphenoid, occipital	Bones and LN	Castration Resistant	133	ADT+Abiraterone / RLT	9.92	Cancer-specific mortality
13	63	Metastatic disease follow-up	R parietal, occipital	Bones	Castration resistant	11.5	RLT	7.23	Cancer-specific mortality
14	79	Metastatic disease follow-up	R parietal	Bones, LN, and visceral (pulmonary)	Castration resistant	5.03	RLT / ADT+Abiraterone	36.47	Cancer-specific mortality
15	77	Metastatic disease follow-up	Occipital, clivus	Bones	Castration resistant	396	ADT+Abiraterone+MDT	24.15	Cancer-specific mortality
16	53	Metastatic disease follow-up	Occipital, temporal	Bones and LN	Castration resistant	366	Cabazitaxel / ADT+Abiraterone	3.22	Cancer-specific mortality
17	73	Metastatic disease follow-up	Clivus	Bones and LN	Castration resistant	2.4	Enzalutamide / RLT / ADT+Docetaxel	27.30	Cancer-specific mortality
18	90	Metastatic disease follow-up	R frontal	Bones, LN, and visceral (liver and adrenal gland)	Castration resistant	81	Lu-RLT	8.90	Cancer-specific mortality

ADT, androgen deprivation therapy; Ac, actinium; BCR, biochemical recurrence; L, left; LN, lymph nodes; NA, not available; Lu, Lutetium; MDT, metastasis directed therapy; mo, months; R, right; RLT, radioligand therapy.

*Age when PSMA PET/CT detected skull metastasis of PCa,.

^ψ^Patient had small cell carcinoma of prostrate cancer.

## Data Availability

Data will be made available by the corresponding author upon reasonable request
